# Using the Recovery Capital Model to Explore Barriers to and Facilitators of Recovery in Individuals with Substance Use Disorder, Psychiatric Comorbidity and Mild-to-Borderline Intellectual Disability: A Case Series

**DOI:** 10.3390/jcm12185914

**Published:** 2023-09-12

**Authors:** Esther Pars, Joanne E. L. VanDerNagel, Boukje A. G. Dijkstra, Arnt F. A. Schellekens

**Affiliations:** 1 Department of Psychiatry, Donders Institute for Brain, Cognition and Behavior, Centre for Neuroscience, Radboud University Medical Center, 6525 GA Nijmegen, The Netherlands; arnt.schellekens@radboudumc.nl; 2 Nijmegen Institute for Scientist-Practitioners in Addiction (NISPA), 6525 HR Nijmegen, The Netherlands; j.e.l.vandernagel@utwente.nl (J.E.L.V.); boukje.dijkstra@ru.nl (B.A.G.D.); 3 Department of Human Media Interaction (HMI), University of Twente, 7500 AE Enschede, The Netherlands; 4 Tactus, Centre for Addiction and Intellectual Disability, 7400 AD Deventer, The Netherlands; 5 Aveleijn, 7622 GW Borne, The Netherlands; 6 Behavioral Science Institute, Radboud University Nijmegen, 6525 GD Nijmegen, The Netherlands; 7 Novadic-Kentron, Addiction Care Centre, 5261 LX Vught, The Netherlands

**Keywords:** recovery capital, comorbidity, addiction, mental illness, intellectual disability

## Abstract

Recovery capital (RC) encompasses the wide range of resources individuals can employ to recover from Substance Use Disorder (SUD). It consists of five subdomains: human, social, cultural, financial, and community RC. Negative recovery capital (NRC) represents the obstacles to recovery. Research on (N)RC in complex multimorbid populations is scarce. This study offers an initial exploration of the viability of (N)RC in three individuals with SUD, psychiatric comorbidities, and an intellectual disability (a triple diagnosis) in inpatient addiction treatment. We collected case file data, ranked recovery goals, and conducted follow-up interviews. The data were subjected to template analysis, using (N)RC domains as codes. All domains were prevalent and relevant, showing dynamic and reciprocal effects, influenced by critical life events acting as catalysts. Notably, during treatment, patients prioritized individual skill development despite challenges in other domains. RC emerges as a valuable concept for mapping recovery barriers and facilitators in individuals with a triple diagnosis, serving as an alternative to the medical model and complementing the biopsychosocial model. It provides a systematic framework to assess critical factors for recovery in complex cases and accordingly align interventions. Future studies should explore the intersections of NRC domains and the dynamic nature of (N)RC to enhance the understanding of the challenges faced by individuals with a triple diagnosis.

## 1. Introduction

In the field of addiction medicine, Substance Use Disorder (SUD) is acknowledged as a complex problem that cannot be attributed to a single cause [1]. The biopsychosocial model, widely utilized in this field, effectively captures this understanding by elucidating the intricate interactions among the biological, psychological, and social factors that influence the development and persistence of addiction [1,2]. In doing so, this model was one of the first to oppose the dominant medical model and acknowledge the importance of addressing the entire individual, rather than just the addiction [3].

However, in recent years there has been a notable shift, both in research and practice, toward the recovery paradigm [4]. This paradigm represents a departure from the traditional focus on cause and cure, by emphasizing the addicted individual’s journey of rebuilding a meaningful life, re-establishing significant social roles, and fostering a positive identity [5]. Within this context, recovery is seen as a transformative process in which individuals actively strive to enhance their well-being, lead a self-directed life, and fulfill their full potential [6]. Although recovery from addiction is frequently understood as an individual endeavor, it is crucial to acknowledge that it is also a social process shaped by contextual factors across multiple levels, such as social networks and societal influences [7]. 

The ecological framework of recovery capital (RC) aids the understanding of how factors in- and outside the individual affect the recovery process [8,9]. RC refers to the total tangible and intangible resources that individuals can employ to recover from SUD [9,10]. Recently, Hennessy [9] mapped the most widely used RC domains: human capital (personal characteristics enabling goal achievement), social capital (resources available through relationships), financial capital (material resources), cultural capital (behavior, norms, and attitudes arising from a cultural group membership that promotes recovery), and community capital (community treatment resources, attitudes, and policies related to recovery). Even though RC’s framework originally focused on resources, incorporating its negative counterpart (negative recovery capital (NRC)) was recently proposed, turning RC into a continuum [11]. NRC refers to recovery-impeding internal and external factors that can occur within the same domains as RC [8,10]. A recovery supporting social network, for example, could be labeled as social RC, whereas a substance abusing social network could be labeled as social NRC. 

Though qualitative and quantitative studies demonstrated the usefulness of RC as a framework to understand the factors affecting recovery, there remain gaps in its conceptual base [9]. First, NRC remains under-researched and inconsistently conceptualized [10]. Second, as the concept of RC and its subdomains was originally developed from studies on individuals in natural recovery (e.g., without treatment), the early conceptualization of RC mainly stemmed from those who already possessed resources and privileges [9]. RC research in less privileged, marginalized populations, as well as populations with multiple clinical diagnoses or in treatment settings is scarce [9,11,12]. Hence, scholars stressed the importance of exploring and critically examining the relevance of the current (N)RC domains for such populations, as RC’s underlying assumptions might not fit with the perceptions and lived experiences of these groups [9,11,12].

The current paper responds to this knowledge gap by examining a particularly vulnerable and under-researched population with SUD, namely, individuals with a triple diagnosis: a combination of SUD, psychiatric comorbid disorders, and a mild-to-borderline intellectual disability (MBID, IQ 50–85). Little is known about recovery and (N)RC in individuals with a triple diagnosis, as most studies focus on dual diagnoses (SUD and MBID *or* mental illness). From these dual diagnosis studies, it is evident that those with co-occurring disorders face a heightened risk of NRC across multiple domains: (a) human NRC (sensitivity to peer pressure, suboptimal coping skills, and lower cognitive and adaptive functioning) [13], (b) social NRC (rejection by family members and a smaller network size) [14,15], (c) financial NRC (housing and employment difficulties) [16], and (d) cultural NRC (stigma) [17]. Further, the scarce existing literature on a triple diagnosis highlights experienced barriers to treatment (e.g., community NRC) that result in (a) reduced access to treatment, (b) a shorter treatment duration, and (c) higher dropout rates [18]. 

Applying the RC framework with all (N)RC domains to persons with a triple diagnosis may aid in (a) a thorough and broader understanding of the issues they face, (b) identifying previously undiscovered RC sources, and (c) directing interventions in treatment and support. The aim of this study is to offer a preliminary exploration of the viability and merit of utilizing the RC model to map barriers to and facilitators for recovery in a complex population with a triple diagnosis by zooming in on three patients with a triple diagnosis, who were admitted to an inpatient SUD treatment program, and mapping (a) their life and medical histories, (b) their recovery goals, and (c) their post-treatment experiences using the RC framework.

## 2. Materials and Methods

### 2.1. Setting and Participants

This case series, as part of a larger research project on recovery of patients with SUD [19], was conducted at a clinic offering inpatient group treatment following the principles of the Community Reinforcement Approach (CRA). Built upon the foundations of operant conditioning, CRA assists individuals in reshaping their daily routines, rendering the choice of a wholesome, drug-free lifestyle more gratifying and effectively competing with alcohol and drug use. Consequently, therapists actively prompt patients to gradually immerse themselves in alternative, substance-free, enjoyable social activities, while endeavoring to heighten the fulfillment experienced within the “communities” of their family, friends, and professional life [20]. As such, the principles of CRA are well-aligned with the RC model. 

Patients admitted to the treatment program encountered multiple problems, including SUD, psychiatric comorbid disorders, MBID, severe social disintegration, a long history of (fragmented) treatment, and care from multiple providers. 

### 2.2. Selection and Procedure

We selected a purposive sample of three patients, based on three criteria: (a) meeting the criteria for triple diagnosis, (b) abundance of pre-treatment data (i.e., full psychological reports, comprehensive intake data), and (c) informed consent to participate in this case series. While meeting the criteria for triple diagnosis (criterion a) often entails a high degree of problem complexity, we aimed to include participants with varying degrees of complexity. The first two criteria proved challenging, as most patients had a long history of many different care providers as well as incomplete or fragmented case files. Three patients met the first two criteria and gave their informed consent.

### 2.3. Data Sources and Collection

We collected data from three different sources to gather a comprehensive view of all (N)RC domains. To examine pre-treatment life events, substance use, treatment episodes, and formal diagnoses, we explored electronic patient case files. To identify priorities in recovery goals, patients participated in multiple assessments during treatment. They were presented with a comprehensive set of 42 clinical, functional, and personal recovery goals, from which they selected the most significant objectives and discussed them together with the researcher (EP). To assess patients’ post-treatment situation, we conducted telephone interviews three months after treatment. In these interviews, we discussed life events, housing, professional help, informal support, wellbeing, coping, stigma, craving, and relapse. Six months post-treatment, the patients were again briefly contacted by phone to determine any changes compared to the earlier interview. 

### 2.4. Analysis

After gathering and transcribing all relevant data, we conducted an in-depth within-case analysis and summarized all individual cases describing their pre-treatment histories, diagnoses, treatment goals, and post-treatment situations. We then conducted a template analysis [21,22]. This flexible technique employs (initial) a priori themes to code and analyze qualitative data, from which a coding template is developed to summarize important themes in the dataset. By using the RC domains as a priori themes (e.g., predefined categories for structuring our data), we delineated the (N)RC domains within our cases and assessed how well the framework fit the data. 

## 3. Results

### 3.1. Case Descriptions

#### 3.1.1. Patient 1

Patient 1, a 40-year-old male, presented with an SUD history of two decades, involving alcohol, cannabis, cocaine, and tobacco. Prior to admission, he used 28 units of alcohol, 2 joints, and 16 cigarettes daily and crack cocaine twice a month. His medical history included an unspecified personality disorder, a mild intellectual disability (total IQ: 66), a mood disorder, alcoholic hepatitis, and a traumatic brain injury with persisting neurocognitive decline. Prescribed medication included citalopram, promethazine, quetiapine, and zopiclone. The family history was positive for alcohol use disorder (the patient’s grandfather).

During his childhood, patient 1 faced expulsion from multiple primary schools before transitioning to specialized primary education for children with severe behavioral problems. He completed vocational education and engaged in blue-collar employment until a traffic accident that caused a traumatic brain injury at the age of 20 years. Following that accident, the patient used alcohol as a coping mechanism for sensory processing difficulties. Prior interventions for patient 1 included outpatient care, ambulatory counseling, pharmacotherapy, sheltered living arrangements, and 14 rounds of inpatient addiction treatment, which did not result in long-term abstinence. Notably, the patient had a limited social network and had associations with substance users, resulting in domestic disturbances and eventually eviction from his residence.

Upon entering treatment, patient 1’s primary objective was to develop assertiveness skills (“standing up for myself”). He acknowledged that the absence of these skills played a pivotal role in both him experiencing bullying and exacerbating his psychological challenges, encompassing feelings of sadness, tension, fear, and excessive worrying. Over time, he expanded his goals to encompass self-acceptance, self-belief, and effective coping strategies. Patient 1 expressed a strong reliance on religion, specifically Christianity, as a source of strength and sought to deepen his understanding of it. Additionally, he emphasized the significance of finding purpose in life, which would contribute to a positive mindset, increased self-worth, the establishment of a healthy routine, and the pursuit of hobbies.

By the third month of treatment, interpersonal conflicts between patient 1 and other residents escalated, leading to an early transfer to a drug-free assisted living facility. This environment provided him with a sense of belonging and support from both peers and staff, promoting social and emotional growth (“I am feeling much stronger and more sociable now”). Despite these positive developments, the patient experienced setbacks, including relapses and selling his medication to another resident. Consequently, he was relocated to an assisted living facility with less strict drug policies. At the six-month follow-up, the patient’s long-term residential arrangements remained uncertain.

#### 3.1.2. Patient 2

Patient 2, a 39-year-old male, presented with a 20-year history of amphetamine, alcohol, tobacco, and cannabis use disorder. His daily substance intake included 1 gram of amphetamine, 2 joints, 20 cigarettes, and 2 units of alcohol. Additional diagnoses included a mild intellectual disability (total IQ 51) and ADHD, for which methylphenidate was prescribed during treatment.

During childhood, patient 2 attended special education for individuals with learning disabilities and later completed vocational education. In his teenage years, he became immersed in a subculture centered around hardcore dance music and raves, in which drug use was highly prevalent. Initially limited to weekends, his substance use escalated to daily stimulant use at the age of 25, after the traumatic experience of finding out his father was deceased. With support from his partner, the patient achieved abstinence at age 35, five years following the birth of his first child. However, two years later, increased work responsibilities combined with performance anxiety triggered occasional stimulant use, which rapidly escalated to daily use.

Patient 2 entered treatment with the primary goal of “becoming a better father”, while also expressing a desire to reduce cravings and develop the skills needed for setting personal goals. He recognized the importance of remaining abstinent, as relapsing could result in “losing everything”. Coping with his addiction was crucial for patient 2, and he also emphasized the importance of working through his past, including the death of his father and his feelings of low self-esteem. In addition, the patient recognized the significance of accepting his learning disability and taking responsibility for his actions. He was determined to learn how to do things independently, such as administrative tasks and opening the mail.

Six months after completing treatment, the patient achieved complete abstinence, successfully resumed employment, and anticipated the arrival of his second child. However, he faced challenges accessing the desired aftercare and expressed that he missed the supportive structure of the clinic. Additionally, his living environment was fraught with drug dealers, and, although his financial situation was stable, he was ineligible for new housing. Initially at risk of relapse due to boredom, patient 2 managed to maintain abstinence by staying engaged in activities, such as attending to household chores and increasing his working hours. Throughout this process, he received invaluable support from his family, employer, and friends. The impending arrival of his second child served as a strong motivator for abstinence.

#### 3.1.3. Patient 3

Patient 3, a 43-year-old female, presented with a 28-year history of alcohol, cannabis, amphetamine, and tobacco use disorder. Her daily consumption included 20 units of alcohol, 20 cigarettes, and 1 joint. Previous substance misuse involved speed, cocaine, and gamma hydroxybutyrate (GHB). She had a medical history that included a cluster-B personality disorder with borderline and antisocial traits, borderline intellectual functioning (total IQ: 74), and ADHD, for which she used methylphenidate. The patient had been incarcerated four times and had undergone one long-term inpatient addiction treatment, resulting in a seven-year period of abstinence. Her family history indicated alcohol use disorder (mother and sister).

During childhood, the patient experienced emotional neglect that worsened after her parents’ divorce, leading her to live with her often-absent alcoholic mother. From the age of 12, she exhibited problematic behavior at school and faced multiple expulsions. Despite these challenges, she managed to complete vocational education and held various jobs until the age of 26. Patient 3 had a history of troubled romantic relationships, with her first boyfriend introducing her to crack cocaine and cannabis. She later found herself involved in several abusive relationships, often resulting in homelessness after break-ups. Two children resulted from these relationships, but the patient lost custody of both children, each time leading to an increase in substance use. Given the choice between incarceration and long-term treatment by a probation officer, she opted for the latter, hoping to have more contact with her children.

Throughout her eight months of inpatient treatment, four recurring goals were identified by the patient. These included (1) maintaining sobriety to (2) avoid legal troubles, (3) reconnecting with her children, and (4) finding suitable housing (necessary for enabling her children to visit her). However, while undergoing treatment, patient 3 faced additional setbacks, as youth care denied contact with her youngest child, leading to a temporary relapse. In addition, finding suitable housing proved challenging due to her delinquent past, as she expressed: “they just don’t want me there”. With the assistance of a social worker, she eventually secured a place in an assisted living facility. Toward the end of her treatment, the patient’s focus shifted toward life beyond the clinic, including paying off debts and seeking (unpaid) employment. Establishing a supportive social network was also important, since she was moving to a new living facility far away from her previous hometown and had limited social connections.

Six months after completing treatment, the patient had returned to the clinic. Referring to her initial stay at the assisted living facility, she described a sense of loneliness and feeling out of place. A lack of daytime activities and the inability to find a job added to her struggles. Within two months of her stay in the assisted living facility, she experienced the loss of three close relatives, further diminishing her social network. Strict rules were imposed due to positive alcohol test results, which she subsequently violated, leading to her expulsion. Following this incident, the patient experienced a full relapse and stayed with a friend until being readmitted to the clinic.

### 3.2. Synthesis of Results

The patients’ case files mainly reported a history of human NRC, such as SUD, various mental health problems, mild-to-borderline intellectual disability (MBID), and a lack of healthy coping mechanisms. The patients themselves, however, did describe human RC factors, such as perceived coping skills and “feeling strong and sociable”. Interestingly, most patients tended to prioritize goals related to human RC, while also experiencing severe challenges in most other domains.

*Social (N)RC* was described in both positive and negative forms. Examples of social RC included ties with family and friends, which strongly motivated both patient 2 and 3 to seek treatment. Social NRC was diverse, spanning past and present, and seemed to be associated with substance use initiation, the emergence of mental health problems, relapse, and recovery stagnation. It included childhood adverse events, substance-abusing parents, abusive relationships, peer pressure, bullying, and substance-abusing or limited social networks.

*Financial (N)RC* was reported in all cases, with patient 2 on the positive (stable income and housing) side and patient 1 and patient 3 on the negative side of the spectrum (unemployment, debts, and homelessness). Obtaining housing was important for patient 1 and patient 3, but finding paid employment was not prioritized over hobbies and volunteer work. For patient 2, paid employment was a source of “ambivalent” RC (e.g., RC and NRC); in addition to being a stable source of income and meaningful daily activities, it also increased stress and triggered relapse. 

Sources of *cultural (N)RC* were implicitly present in all three cases. RC included culturally informed perceptions of “good parenting”, religion, and being embedded in a recovery-promoting subculture while in treatment. NRC included being part of a substance-use-promoting subculture and MBID stigma.

*Community (N)RC* was frequently mentioned, in both positive and negative terms. Various institutions and organizations enabled patients to receive professional guidance, treatment, and access to assisted and sheltered housing. However, the transition between different treatment settings, including the shift from inpatient to outpatient care or from a clinic to sheltered or assisted housing, did not always occur smoothly. Such “bumpy” transitions became a source of community NRC, as was observed in all three cases. This form of NRC was especially impactful when housing and care were intertwined, for example, by placing patients in an abstinence-contingent sheltered living facility, where they faced the risk of losing both their housing and current care in the event of relapse (Table 1).

## 4. Discussion

To our knowledge, this study is the first to preliminary explore the viability, merit, and application of the RC framework in a population with Substance Use Disorder, psychiatric comorbidity, and a mild-to-borderline intellectual disability (i.e., a triple diagnosis). Using this model, we were able to aptly capture and structure the different factors facilitating and impeding recovery in our three exemplary cases. Below, we first reflect on the (N)RC domains as they applied to our cases, after which the viability and merit of the model for this population are discussed. 

### 4.1. Human (N)RC

While all RC domains were represented in our cases, mentions of human (N)RC were particularly present in the case files and patient’s recovery goals. The case files mainly reported on negative human RC, which is in line with the scientific literature describing individuals with MBID, SUD, and/or psychiatric comorbid disorders as a vulnerable, at-risk population [13,18,23]. As such, these enumerations of negative human RC could be seen as a typical example of the risks and vulnerabilities this population faces. Alternatively, they can be interpreted as an expression of what Treloar and Holt [24] coined the “deficit model”, in which those seeking SUD treatment are mostly described as “problems to be solved”. Within this context, it is particularly interesting that two patients tended to prioritize obtaining human RC, despite also experiencing severe challenges in most other domains. A study on NRC by Gavriel-Fried and Lev-El [10] in individuals with a gambling disorder yielded similar tendencies, potentially indicating high levels of awareness of a patient’s own role in their addiction. However, the authors cautioned that prioritizing human RC could also indicate a perception of addiction recovery as an individual responsibility rather than a shared societal one. 

### 4.2. Social (N)RC

In our cases, social RC seemed to boost recovery, whereas social NRC was associated with substance use initiation, relapse, and recovery stagnation. These compelling effects of social ties and support of recovery are also extensively described in the literature [25,26]. For example, being responsible for children and maintaining ties with family and friends were identified as strong motivators for abstinence—also in marginalized, complex populations [27]. Social support was also related to recovery, abstinence, and treatment retention [26]. Contrastingly, negative social influences, such as a childhood adversity (like emotional neglect) and substance-abusing social networks, are linked to substance use and relapse [28,29]. Furthermore, having SUD, MBID, or a mental illness is associated with fewer social support network resources and a lesser quality and quantity of social networks [30,31,32]. Indeed, two out of three cases (patient 1 and patient 3) had limited or declining social networks. Only one (patient 2) had access to a supportive, recovery-facilitating social network, which even seemed to compensate for a lack of fitting aftercare (e.g., community NRC). 

### 4.3. Financial (N)RC

In our cases, financial (N)RC ranged from positive (stable income and housing) to negative (unemployment, debts, and homelessness). The latter is in line with studies stating that individuals with SUD face an increased risk of debt [33], and those with mental illness and MBID are, compared to the general population, less likely to find employment or adequate housing [34]. While housing was important to those with financial NRC, hobbies or volunteer work were valued over paid employment, indicating that being engaged in meaningful activities was more important than financial gains [35]. The latter, however, should be interpreted within the Dutch context, where social benefits provide for basic needs.

### 4.4. Cultural (N)RC

Cultural (N)RC is the most implicit domain, yet, in our cases, it nonetheless appeared influential. At the intersection of cultural and social RC, culturally informed perceptions of “good parenting” (that is, sober parenting) prompted patients to engage in inpatient treatment. As such, the stigma associated with parental substance use [36] was converted into a motivational force. Another mentioned source of cultural RC, religion, is firmly supported by the literature, describing religion or spirituality as a contributor to recovery [37]. A final source of cultural RC was being embedded in a recovery-promoting subculture while in treatment, which facilitates the transition from a “culture of addiction” to a “culture of recovery” [38]. 

NRC also presented itself at the intersection of the social and cultural, as some patients mentioned that substance use initiation occurred while being part of a substance-use-promoting subculture. This association is confirmed in the literature, which states that subculture affiliation and substance use are significantly linked [39]. A final source of cultural NRC was perceived MBID stigma, which served as a catalyst for substance use. According to Slayter, [18], individuals with MBID are indeed more likely to have experienced stigma, which increases the risk of SUD. 

Finally, cultural (N)RC may also partly explain patients’ prioritization of human RC. As mentioned, this might be informed by an individualized take on recovery, which is common in neoliberal societies valuing human RC factors, such as self-management, rationality, and choice, rather than societal responsibility [40,41,42].

### 4.5. Community (N)RC

Various institutions and organizations enabled patients to receive professional guidance, treatment, and access to assisted and sheltered housing. Yet, as professional help or treatment settings were not always fitting, community RC could turn into community NRC, which was especially impactful when abstinence-contingent housing and care were intertwined. While the latter reduces the relapse risks associated with substance use exposure [43], it also increases the risk of housing instability once relapse occurs [44]. Multimorbid populations with SUD may, thus, face distinct challenges at the intersection of the financial (housing) and community (care) (N)RC domains. Frictions among patients’ treatment needs and the available care are frequently mentioned specifically for those with SUD and MBID. ID services generally lack the skills to treat SUD, whereas addiction services are not equipped to guide those with MBID [18,45,46,47]. 

### 4.6. Interaction of Domains

Our findings suggest that, especially for complex cases, the convergence between several different NRC factors (for example patient characteristics, housing, and care) can have serious consequences and should, therefore, be explored in more detail. Our data also imply that, for our complex cases, the (N)RC domains are not static, show reciprocal effects [10], and are influenced by critical life events that may serve as a catalyst [27]. Indeed, throughout the life trajectories of these three cases with a triple diagnosis, we observed how, at vital moments, and through the interaction between life events and (N)RC domains, recovery processes gained momentum—or lost their bearing. By adopting a dynamic approach to RC, the model seems adequately able to holistically capture recovery barriers and facilitators in complex populations with a triple diagnosis. 

### 4.7. On the Added Value of RC

Examining three cases with a triple diagnosis through the lens of RC provided us with an initial, comprehensive, and well-demarcated understanding of the factors that contribute to or hinder recovery. Such insights hold significant value for both research and clinical practice, particularly when addressing the complex challenges faced by this population, which can be daunting for patients, researchers, and healthcare professionals alike. As such, the RC framework serves as an effective antidote to one-sided focus or deficit models by moving beyond the predominant emphasis on the individual and community (N)RC in the existing literature on (dual and) triple diagnoses.

Moreover, the RC model holds promise in guiding the determination of the appropriate level of care. By considering the areas of concern *and* strengths that individuals bring to SUD treatment, personalized care can be provided. For example, the recovery capital/problem severity matrix, as proposed by White and Cloud [23], takes into account the complexity and severity of problems as well as the level of available recovery capital, in order to determine the optimal level of care. Such approaches facilitate that treatment plans aligns with each individual’s unique needs and resources, maximizing the likelihood of successful recovery.

Future studies might consider applying the RC model to assess a series of real-world patients with a triple diagnosis, which allows for exploring how an RC-based approach may contribute to shaping practical care for these patients and the development of appropriate treatment systems. Existing tools, such as the Brief Assessment of Recovery Capital (BARC-10) [48] or the more comprehensive Assessment of Recovery Capital (ARC) [49], can be used in this endeavor. This could strengthen the integration of the RC framework into both clinical practice and research amongst the most vulnerable populations. 

### 4.8. Strengths and Limitations

This case series is the first to preliminary explore RC in individuals with a triple diagnosis, thereby adding to the scarce information available about RC in this population. We demonstrate the merits of adopting a comprehensive approach to recovery, instead of a narrow individual focus. However, due to the small sample size and the lack of available literature on triple diagnoses, we cannot make solid claims about the larger population with a triple diagnosis or about the causal relationships between a triple diagnosis and RC domains. More research is warranted to explore this further. 

It is worth emphasizing that, although all participants were admitted to the same clinic and participated in a CRA-based program, they were exposed to different treatment modalities before, during, and after treatment. In this exploratory study, we did not control for potential effects. Instead, this diversity enabled us to investigate different manifestations of community (N)RC, particularly in various treatment contexts. Additionally, it is important to note that the RC framework closely aligns with the CRA approach offered at the clinic. While this alignment might introduce bias by increasing RC post-treatment, it is crucial to stress that this effect was not consistently observed in at least two out of the three patients during the follow-up period. This suggests that bias was limited. Bias in individuals’ perception of their recovery capital due to CRA treatment, however, cannot be fully ruled out. Yet this may also apply to other interventions such as cognitive behavioral therapy, motivational interviewing, and self-help groups like AA or the 12-step approach and, thus, not be unique to CRA.

Finally, we did not explicitly examine whether new RC domains could be revealed through our template analysis. This might be a topic for further research among a larger sample with a triple diagnosis, in which our contribution to the RC framework—and beyond—can be expanded further. Future research should prioritize investigating the inter-relationships among NRC domains and the dynamic nature of (N)RC, in order to enhance our understanding of the unique challenges faced by *and* the resilience exhibited by individuals with a triple diagnosis.

## 5. Conclusions

The concept of recovery capital represents a promising and practical framework for analyzing both the barriers to and facilitators of recovery in complex populations. It provides a compelling alternative to one-sided or deficit-based approaches, and it may provide valuable assistance to practitioners to determine the appropriate focuses of care for our most challenging populations.

## Figures and Tables

**Table 1:** Template for recovery capital in three cases with triple diagnosis.

Human (N)RC	Social (N)RC	Financial (N)RC	Cultural (N)RC	Community (N)RC
Feeling strong/sociable (+) Coping skills (+/−) SUD (−) Mental health problems (−) MBID (−) Low self-esteem (−)	Supportive partner/friends (+) Family ties (+) Custody loss (−) Abusive romantic relationships (−) Substance using parents/friends (−) Peer pressure (−) Bullying (−) Limited social network (−)	Adequate housing (+) (Un)paid employment (+/−) Homelessness (−) Unemployment (−) Debts (−)	Recovery promoting subculture in treatment (+) Perceptions of “good parenting” (+) Religion (+) Substance use promoting subculture (−) MBID stigma (−)	Professional (post)treatment and guidance (+/−) Access to (sheltered/assisted) housing (+/−) Criminal record and probation (+/−)
+ RC − NRC				

## Data Availability

Not applicable.
